# Effects of immunonutrition on biomarkers in traumatic brain injury patients in Malaysia: a prospective randomized controlled trial

**DOI:** 10.1186/s12871-017-0369-4

**Published:** 2017-06-15

**Authors:** Vineya Rai Hakumat Rai, Lee Fern Phang, Sheau Fung Sia, Amirah Amir, Jeyaganesh S. Veerakumaran, Mustafa Kassim Abdulazez Kassim, Rafidah Othman, Pei Chien Tah, Pui San Loh, Mohamad Irfan Othman Jailani, Gracie Ong

**Affiliations:** 1 School of Medicine, Taylor’s University, Lakeside Campus, 47500 Subang Jaya, Malaysia; 2 KPJ Tawakkal Specialist Hospital, Jalan Pahang Barat, 53000 Kuala Lumpur, Malaysia; 3 Department of Anaesthesiology, Hospital Umum Sarawak, Jalan Hospital, 93586 Kuching, Sarawak Malaysia; 4 Division of Neurosurgery, Faculty of Medicine, University of Malaya, 50603 Kuala Lumpur, Malaysia; 5 Department of Parasitology, Faculty of Medicine, University of Malaya, 50603 Kuala Lumpur, Malaysia; 6 Department of Anaesthesiology, Faculty of Medicine, University Malaya, 50603 Kuala Lumpur, Malaysia; 7 Department of Dietetics, University Malaya Medical Centre, 50603 Kuala Lumpur, Malaysia

**Keywords:** Biomarkers, Immunonutrition, Traumatic brain injury, Neomune, Enteral nutrition, Cytokines

## Abstract

**Background:**

Head injury is one of the top three diagnosis leading to intensive care unit (ICU) admission in Malaysia. There has been growing interest in using immunonutrition as a mode of modulating the inflammatory response to injury or infection with the aim of improving clinical outcome. The aim of the present study was to evaluate the effect of an immunonutrition on biomarkers (IL-6, glutathione, CRP, total protein and albumin) in traumatic brain injury patients.

**Methods:**

Thirty six patients with head injury admitted to neurosurgical ICU in University Malaya Medical Centre were recruited for this study, over a 6-month period from July 2014 to January 2015. Patients were randomized to receive either an immunonutrition (Group A) or a standard (Group B) enteral feed. Levels of biomarkers were measured at day 1, 5 and 7 of enteral feeding.

**Results:**

Patients in Group A showed significant reduction of IL-6 at day 5 (*p* < 0.001) with concurrent rise in glutathione levels (*p* = 0.049). Patients in Group A also demonstrated a significant increase of total protein level at the end of the study (day 7).

**Conclusion:**

These findings indicate the potential of immunonutrition reducing cytokines and increasing antioxidant indices in patients with TBI. However, further studies incorporating patient outcomes are needed to determine its overall clinical benefits.

**Trial registration:**

National Medical Research Register (NMRR) ID: 14–1430-23,171. ClinicalTrials.gov identifier: NCT03166449.

## Background

In 2014, injury ranked fifth as the most common cause of hospitalisation in Malaysia, with 86% of major trauma patients sustaining injuries to head and neck [1, 2]. It is known that injuries to the brain is among the most likely to result in death and disability [3]. Therefore, it was unsurprising when the Malaysian Registry of Intensive Care reported head injury as one of the top three diagnosis leading to intensive care unit (ICU) admissions with an in-hospital mortality rate of 22.1% [4]. In addition to causing a significant problem in Malaysia, injury related mortality and morbidity also affects countries worldwide and is expected to be a major cause of death and disability by the year 2030 [5].

Patients with traumatic brain injury (TBI) often develop a hypermetabolic state whereby there is an increase in energy expenditure [6–8]. Consequently, this led to clinicians acknowledging nutritional support as an essential component in managing critically ill patients [9]. A recent meta-analysis showed that early nutrition in patients sustaining TBI was associated with a significant reduction in mortality rate, lower risk of infectious complications and lower risk of poor outcome [10].

Due to its dynamic pathophysiology, the damage in TBI does not stop following the primary insult. A combination of systemic derangements (hypoxia, hypotension or hypercarbia) and local events (inflammation, oxidative stress and ion imbalance) often lead to secondary brain injury [11]. There has been growing interest in using immunonutrition as a mode of modulating the inflammatory response to injury or infection with the aim of improving clinical outcome [12, 13]. Immune-enhancing or immune-modulation enteral formula has been shown to significantly reduce infection rate in TBI patients [10, 14–16]. In fact, a systemic review involving critically ill patients showed that the use of immune-modulating diets supplemented with fish oil improved the outcome of medical ICU patients (with SIRS/sepsis/ARDS) [17]. Omega-3 fatty acids namely docosahexaenoic acid (DHA) and eicosapentaenoic acid (EPA) found in fish oil are thought to play an important role in the repair and resolution of inflammation [18]. These two fatty acids also boost immune response by improving lymphocyte function [19]. Interestingly, research on DHA and EPA using animal model have shown favourable outcome in its neuro-restorative capacities in TBI [20].

This study aims to evaluate the effect of a specific immunonutrition, Neomune, on biomarkers (cytokines, acute phase serum proteins and antioxidants) in traumatic brain injury patients. This immune enhancing enteral feed contains arginine, glutamine and omega-3 fatty acid.

## Methods

This study and its protocol was approved by the Medical Ethics Committee University Malaya Medical Center (MEC ID NO: 20,143–15). Written informed consent was obtained from patient’s next of kin.

A prospective randomized controlled trial in patients with head injury comparing two high energy protein entera formula; Neomune (manufactured by Thai Otsuka Pharmaceutical Co., Ltd., Thailand) and Fresubin® HP (manufactured by Fresenius Kabi, Bod Hamburg, Germany) was conducted. Fresubin® HP energy is the standard enteral feeding used in neurosurgical ICU patients in University Malaya Medical Centre. Neomune was chosen to study its immune-modulating effects.

Patients receiving Neomune were classified as Group A (*n* = 18), whereas those receiving Fresubin® HP energy as Group B (*n* = 18). Neomune is enriched with arginine, glutamine and omega-3 fatty acid. The composition of these two formulas are described in Table 1.

**Table 1:** Composition of enteral formula

Formula	Neomune (1.5 kcal/mL)	Fresubin HP Energy (1.5 kcal/mL)
Classification	Immune-enriched, lactose and gluten free	High energy protein with medium chain triglyceride (MCT), fibre free, lactose and gluten free
Total Energy	1503 kcal	1502 kcal
Carbohydrate	187.5 g/L = 750 kcal (50%)	170 g/L = 680 kcal (45%)
Protein	93.75 g/L = 375 kcal (25%)	75 g/L = 300 kcal (20%)
Fat	42.0 g/L = 378 kcal/L (25%)	58 g/L = 533 kcal (35%)
Protein source (%)	Casein 70% Arginine 20% Glutamine 10%	Casein >90%
Fat source (%)	Corn oil 30%	MCT oil 57%
	Fish oil 20%	Fish oil 0.86%
	MCT oil 50%	
NPC: N	75: 1	100: 1

### Subject recruitment criteria

Thirty six patients with head injury admitted to neurosurgical ICU, University Malaya Medical Centre were recruited for this study, over a 6-month period from July 2014 to January 2015. Patient’s inclusion criteria were a) age between 15 and 78 years old, b) admission within 48 h of post traumatic event, c) moderate to severe head injury (Glasgow Coma Scale 3–12) and, d) requiring enteral nutrition. Exclusion criteria were a) history of uncontrolled diabetes mellitus, b) history of renal or liver dysfunction, c) severe sepsis with multi organ failure and d) history of significant abdominal or chest injuries requiring major surgery.

### Feeding protocol

Eligible patients were randomized into receiving either Neomune (Group A) or Fresubin® HP energy (Group B) using a computerized random number generator. Fresubin® HP energy is a premixed formula with an energy of 1.5 kcal/ml. Neomune formula comes in powder form and thus was prepared to reach a concentration of 1.5 kcal/ml in order to make it comparable for the trial.

Enteral feeding was delivered within 24 to 48 h after admission or surgery according to Brain Trauma Foundation (BTF) Guidelines [21]. All feeding were introduced via nasogastric tube and carried out using infusion pump. Enteral feeding was commenced at an initial rate of 20 ml/h, and increased by 20 ml/h every 6 h until target calorie was reached, provided that there was no significant gastric residual volume (<300 mL). The target calorie for each patient was determined by the clinician working alongside the dietitian using Harris Benedict equation which measures Resting Energy Expenditure (REE). This was further corrected 140% of REE based on studies done on traumatic brain injured patients [22, 23] and nutrition guidelines published by the BTF, the American Association of Neurological Surgeons, and the Joint Section on Neurotrauma and Critical Care [21]. Patients going for scheduled surgery are kept nil by mouth 6 h prior surgery. Feeding interruption are also kept to a minimal if patients are undergoing procedures, extubation or imaging.

### Data collection

Venous blood was withdrawn from patients at day 1, 5 and 7 of enteral nutrition to measure the levels of interleukin-6 (IL-6), glutathione, C-reactive protein (CRP), total protein and albumin. The concentration of IL-6 and glutathione were determined by ELISA (R&D Systems, Minneapolis, USA) according to manufacturer’s protocol. Prealbumin was not included as a nutritional biomarker in this study because this test is not available in our standard laboratory test. Furthermore, we were also limited by resource constraint. All measured outcomes were compared between Group A and Group B.

### Sample size analysis

The sample size for this study was calculated based on a similar study done previously on immune enhancing nutrition in traumatic brain injury by Painter et al. [24]. The following formula was applied:

$$ \boldsymbol{n}=\frac{2{\boldsymbol{\sigma}}^2{\left(\frac{\boldsymbol{Z}\boldsymbol{\alpha }}{2}+\boldsymbol{Z}\boldsymbol{\beta } \right)}^2}{{\mathrm{\triangle}}^2} $$

where *n* is the required sample size. The symbol σ represents the standard deviation of the outcome variable. Painter et al., found that the mortality rate for patients receiving immune enhancing nutrition formula in TBI was 7.5% (0.075) and thus, this value was used as σ in the above calculation [24]. ∆ symbolizes the effect size, Zα/2 represents the desired level of statistical significance whereas Zβ represents the desired power. In this study, we determined our effect size as 0.05 as the standard value for detectable difference, confidence interval as 95% (Zα/2 = 1.96) and power of 80% (Zβ = 0.84). The final sample size obtained was 35.28. A total number of 36 patients were used in this study.

### Statistical analysis

The data were analysed using SPSS (Statistical Package for the Social Sciences) software version 20.0. Paired t-test was used to compare the concentration of cytokines, acute phase serum proteins and antioxidants between day 1, 5 and 7 after the administration of enteral feeding. Independent sample t-test was used to compare continuous data which include cytokines, acute phase serum proteins and antioxidant levels between Group A and Group B. Statistical significance was predetermined at *p* < 0.05.

## Results

### Demographic and clinical characteristics

Demographic and clinical characteristics were obtained from all 36 patients as displayed in Table 2. Mean age in Group A and Group B was 42 ± 70 (range: 15–75) and 32 ± 50 (range: 15–56) years old respectively, with male predominating in both groups. The main cause of brain injury in this study were a consequence of motor-vehicle accident, with more than half of patients in each group being admitted with severe Glasgow Coma scale (GCS) score (<8). There were no significant differences between Group A and Group B with respect to demographic data, GCS scoring, diagnosis and clinical interventions.

**Table 2:** Demographic profile and clinical data of patients in Group A and B

Parameter	Enteral feed		*p* value
	Group A	Group B	
	(*n* = 18)	(*n* = 18)	
Gender (%):			0.560
Male	94.4	88.9	
Female	5.6	11.1	
Age [mean] (years)	42	32	0.067
Weight [mean] (kg)	68.4	70.6	0.533
Mode of injury (%):			0.445
Motor-vehicle accident	66.7	83.3	
Fall	27.8	11.1	
Assault	5.6	5.6	
Diagnosis (%):			0.972
Subarachnoid haemorrhage	16.7	22.2	
Subdural haemorrhage	33.3	27.8	
Extradural haemorrhage	16.7	16.7	
Mixed haemorrhage	33.2	33.3	
Glasgow Coma Score (%)			0.278
Minor	0	0	
Moderate	22.2	38.9	
Severe	77.8	61.1	
Type of surgery (%):			0.409
Craniotomy	5.6	11.1	
Craniectomy	22.2	11.1	
Ventriculostomy	38.9	61.1	
Conservative	33.3	16.7	

### Effects of immunonutrition on cytokines, acute phase serum proteins and antioxidants indices

Observation in group A from day 1 to 7 showed a downward trend of IL-6 concentration (Table 3). This reduction was significant at day 5 and 7 (Table 4). On the other hand, glutathione and total protein concentration increased from day 1 to 7 which reached significance at day 5 and 7 respectively (Tables 3 and 4). Although there were changes in cytokines, acute phase serum proteins and antioxidants indices in Group B throughout the study, its trend were not consistent and none of the changes were significant.

**Table 3:** The mean of cytokines, acute phase serum proteins and antioxidants concentrations at day 1, 5 and 7

Parameters	Group A	Group B	*p*-value
	Mean (SD)	Mean (SD)	
IL-6 (pg/mL)			
Day 1	113.10 (81.140)	101.90 (76.163)	0.753
Day 5	62.10 (69.141)	89.58 (80.487)	0.248
Day 7	42.18 (39.833)	106.50 (92.992)	0.004 *
Glutathione (μmol/L)			
Day 1	182.00 (100.796)	144.70 (92.958)	0.211
Day 5	245.60 (134.536)	141.60 (55.924)	0.009*
Day 7	270.10 (155.392)	125.20 (79.603)	0.011*
CRP (mg/dL)			
Day 1	10.42 (4.682)	10.96 (6.143)	0.994
Day 5	10.32 (9.348)	12.40 (9.001)	0.368
Day 7	8.31 (9.264)	11.74 (7.425)	0.109
Albumin (g/L)			
Day 1	32.44 (5.469)	34.44 (6.090)	0.303
Day 5	34.06 (6.743)	33.67 (4.325)	0.981
Day 7	35.11 (7.053)	33.11 (5.529)	0.355
Total protein (g/L)			
Day 1	57.17 (7.755)	59.11 (9.424)	0.536
Day 5	62.50 (9.599)	60.50 (5.205)	0.505
Day 7	65.89 (9.875)	62.50 (7.778)	0.232

*p-value <0.05

**Table 4:** The mean difference of cytokines, acute phase serum proteins and antioxidants concentrations between day 1, 5 and 7

Parameters	Group A		Group B	
	Mean difference (SEM)	*p*-value	Mean difference (SEM)	*p*-value
IL-6 (pg/mL)				
Day 1–5	48.49 (10.68)	<0.001*	10.68 (17.78)	0.556
Day 5–7	0.22 (14.29)	0.988	27.19 (20.48)	0.214
Day 1–7	46.73 (19.29)	0.034*	12.55 (34.14)	0.722
Total Glutathione (μmol/L)				
Day 1–5	63.58 (29.98)	0.049*	3.16 (18.33)	0.865
Day 5–7	24.29 (21.05)	0.269	14.08 (22.13)	0.538
Day 1–7	83.41 (39.27)	0.053	2.56 (30.00)	0.934
CRP (mg/dL)				
Day 1–5	1.1 (2.81)	0.701	2.39 (3.18)	0.467
Day 5–7	1.74 (1.43)	0.240	2.06 (1.74)	0.257
Day 1–7	2.47 (2.98)	0.420	1.81 (3.26)	0.590
Albumin (g/L)				
Day 1–5	1.61 (1.71)	0.359	0.78 (1.30)	0.558
Day 5–7	1.06 (1.11)	0.355	0.56 (1.19)	0.645
Day 1–7	2.67 (2.03)	0.207	1.33 (1.52)	0.391
Total protein (g/L)				
Day 1–5	5.33 (2.93)	0.087	1.39 (2.20)	0.537
Day 5–7	3.39 (2.05)	0.117	2.00 (1.82)	0.286
Day 1–7	8.72 (3.26)	0.016*	3.39 (2.25)	0.151

*p-value <0.05

Comparison between both groups revealed significantly lower IL-6 levels at day 7 of receiving enteral feeding in Group A (Table 3). Additionally, glutathione levels in group A at day 5 and 7 were significantly higher compared to Group B (Table 3).

## Discussion

Protein requirements in trauma patients are proportionately higher and thus are not met by provision of routine enteral formula [25]. The appropriate and safe level in the critically ill or hypermetabolic patient in which a pro-inflammatory state exists is much more difficult to determine. Therefore, the enteral formulas used in this study are both of high protein content to meet the BTF recommendation of providing a formula containing at least 15% of calories as protein [21].

This study showed that patients receiving Neomune, an immunonutrition, had significant reduction in their IL-6 levels compared to patients in Group B. Similar observations were reported when immunonutrition was administered to patients with TBI, patients undergoing major hepatobiliary resection and patients with gastrointestinal cancer [16, 26, 27]. Following TBI, IL-6, a key regulator of the inflammatory response, rises in brain tissue [28], cerebrospinal fluid and serum [29]. This cytokine plays a dual opposing role as it is associated with both pro and anti-inflammatory effects [30]. Previous research on TBI serum IL-6 have shown contradicting findings. Kalabalikis et al. reported that there was no association between IL-6 levels with neurological outcome [31]. Whereas, others have demonstrated that high IL-6 level is correlated with poor outcome [32, 33]. For this reason, some authors have suggested reducing inflammatory mediators as a therapeutic strategy for TBI [34, 35]. An animal study showed that the presence of IL-6 may be beneficial during early stages of TBI and therefore, suggested that a timely, targeted approach is recommended in improving outcomes following TBI [36]. Another study involving animals with mild TBI showed that the neutralization of IL-6 resulted in restoration of normal motor coordination and it also mitigates the adverse effects of hypoxia which include exacerbated brain inflammation and injury [37]. Although subject to further studies, we postulate that the outcome from the use of immunomodulation in TBI can be extrapolated to humans and this concept can be exploited using immunonutrition to lower IL-6 levels in order to prevent secondary brain injuries.

This present study also demonstrated a significant increase of glutathione levels in patients receiving Neomune. Since glutamine is an important substrate for glutathione, depletions in glutamine will be reflected by glutathione reduction [38, 39]. In patients experiencing physiological stress, the synthesis of glutamine, a non-essential amino acid is unable to cater to the increased glutamine requirement [40]. Furthermore, patients with brain injury have been observed to experience profound hypoglutaminemia [41–44]. Glutathione has important function as anti-oxidant as it is a scavenger for reactive oxygen species [17]. During the acute ischemic phase of TBI, a hypermetabolic state leads to hyperglycaemia and intracellular lactate production which is associated with the development of reactive oxygen species [45–48]. Thus, tissues depleted of glutathione may undergo oxidative stress leading to complications such as predisposing patients to bacterial translocation and recurrent sepsis [49, 50]. Glutamine supplementation has been associated with lower mortality rates, shorter hospital stay and decreased occurrence of pneumonia and stress ulcer [51].

Patients receiving Neomune demonstrated a significant increase of total protein level at the end of the study whereas no significant change was seen in albumin level throughout the study. Although serum protein and albumin were previously suggested as biomarkers for malnutrition [52], serum albumin is not well correlated to protein mass [53] and does not always significantly increase following nutritional programmes [54]. Whilst albumin and protein level may be useful in the assessment of malnutrition [53], these biomarkers are not helpful at monitoring potential therapeutic effects of nutrition [55].

Immunonutrition came into existence as part of an immune-modulating strategy to improve patient outcome in the critically ill. Many clinical trials, meta-analyses and systemic reviews have been done on the use of immunonutrition in diversed population with inconsistent outcomes [56]. One review on the use of immunonutrition showed that it may reduce infectious complications in trauma and perioperative patients [57], whereas another meta-analyses revealed no effect of immune modulating diets on mortality or length of stay [17]. Other systematic reviews looking at the efficacy of immunonutrition in the critically ill generally concluded that the incidence of infection and hospital length of stay are reduced, with no reduction in mortality [56, 58, 59]. Additionally, another meta-analysis showed that glutamine and selenium supplementation may result in reduction of nosocomial infections among the critically ill [60]. Although it is postulated that immunonutrition influence immune parameters and has anti-inflammatory effects, it is has not always been associated with better clinical outcomes. Some researchers propose that immunonutrition may be harmful in some subgroups of critically ill patients [55]. Rathmacher et al. found that blood urea was raised in patients receiving immunonutrition and attributed this to the combination of increased nitrogen load and the specific effect of arginine and glutamine in stimulating ureagenesis [61]. As such, careful assessment may be needed in patients with kidney and liver disease before immunonutrition can be prescribed. There is an emerging paradigm shift to move from immunonutrition to pharmaconutrition, where effect of individual immunonutrients are assessed independent of standard nutritional support [62]. The way forward is to design future studies similar to drug trials, which include evaluating clinically relevant outcome in a well-defined population [63].

Interestingly, immunomodulators have also been used in combination with antimicrobial therapy in clinical trials in search of new strategies for the treatment of bacterial infections [64]. Unfortunately, the use of immunonutritian was found not to be encouraging the antimicrobial therapy in bacterial infections [65–67]. However, this is an intelligent use of immunomodulators and this idea can be explored to look at other usage of immunonutrition.

This study has some limitations which have to be pointed out. Due to resource constraint, other cytokines indices such as tumour necrosis factor, IL-1, IL-8 and IL-10 were not measured. Confounding factors that may affect biomarker levels such as sepsis was also not included during data collection. Since patients’ overall progress and outcome were not looked into in this study, no definite correlation can be made between immunonutrition and patient outcome.

## Conclusions

The present study showed that IL-6 levels were significantly reduced whereas glutathione levels were significantly higher following feeding with Neomune, an immunonutrition. These findings indicate the potential of immunonutrition reducing cytokines and increasing antioxidant indices in patients with TBI. However, further studies incorporating patient outcomes are needed to determine its overall clinical benefits.

## Abbreviation

ARDS: Acute respiratory distress syndrome
ICU: Intensive care unit
SIRS: Systemic inflammatory response syndrome
TBI: Traumatic brain injury
